# Single-Cell Gene Expression Analysis and Evaluation of the Therapeutic Function of Murine Adipose-Derived Stromal Cells (ASCs) from the Subcutaneous and Visceral Compartment

**DOI:** 10.1155/2018/2183736

**Published:** 2018-12-11

**Authors:** Dominik Pförringer, Matthias M. Aitzetmüller, Elizabeth A. Brett, Khosrow S. Houschyar, Richard Schäfer, Martijn van Griensven, Dominik Duscher

**Affiliations:** ^1^Klinikum rechts der Isar, Technische Universität München, Klinik und Poliklinik für Unfallchirurgie, München, Germany; ^2^Klinikum rechts der Isar, Technische Universität München, Klinik für Plastische Chirurgie und Handchirurgie, München, Germany; ^3^BG Unfallklinik Bergmannsheil, Klinik für Plastische Chirurgie, Bochum, Germany; ^4^Institut für Transfusionsmedizin, DRK Blutspendedienst Baden-Württemberg-Hessen gGmbH, Klinikum der Goethe Universität Frankfurt, Frankfurt, Germany; ^5^Kepler Universitätsklinikum, Johannes Kepler Universität, Linz, Austria

## Abstract

**Introduction:**

Adipose-derived stromal cells (ASCs) are a promising resource for wound healing and tissue regeneration because of their multipotent properties and cytokine secretion. ASCs are typically isolated from the subcutaneous fat compartment, but can also be obtained from visceral adipose tissue. The data on their equivalence diverges. The present study analyzes the cell-specific gene expression profiles and functional differences of ASCs derived from the subcutaneous (S-ASCs) and the visceral (V-ASCs) compartment.

**Material and Methods:**

Subcutaneous and visceral ASCs were obtained from mouse inguinal fat and omentum. The transcriptional profiles of the ASCs were compared on single-cell level. S-ASCs and V-ASCs were then compared in a murine wound healing model to evaluate their regenerative functionality.

**Results:**

On a single-cell level, S-ASCs and V-ASCs displayed distinct transcriptional profiles. Specifically, significant differences were detected in genes associated with neoangiogenesis and tissue remodeling (for example, Ccl2, Hif1*α*, Fgf7, and Igf). In addition, a different subpopulation ecology could be identified employing a cluster model. Nevertheless, both S-ASCs and V-ASCs induced accelerated healing rates and neoangiogenesis in a mouse wound healing model.

**Conclusion:**

With similar therapeutic potential in vivo, the significantly different gene expression patterns of ASCs from the subcutaneous and visceral compartments suggest different signaling pathways underlying their efficacy. This study clearly demonstrates that review of transcriptional results in vivo is advisable to confirm the tentative effect of cell therapies.

## 1. Introduction

The wide-ranging applications of stem cells in regenerative medicine have been scientifically described for decades [1]. Far more than a quarter of a million references will be listed on PubMed in the spring of 2018 alone on the subject of stem cells. In particular, the vast potential of adipose-derived stromal cells (ASCs) from adipose tissue is extensively described [2]. Due to their multiple therapeutic functions, ASCs are attracting increasing attention, especially for their paracrine activity [3] as well as their capacity for differentiation and their potential for use in tissue engineering [4]. The spectrum ranges from cardiac regeneration after infarction [5] via the neovascularization of wounds [6], the regeneration of nerves [7], and the healing of neurological systemic diseases [8] to the treatment of osteoarthritis [9]. ASCs are also an option for tissue regeneration after adjuvant radiotherapy [10].

The functionality of (re) implanted cells is influenced by a variety of factors whose exact interactions are the subject of numerous studies [4]. In the context of cell-assisted fat transplantation, it should be noted that the dose of the introduced cells has a significant influence on the survival of the transplant [11]. In addition, it has been shown that cellular heterogeneity has an effect on the healing potential of stem cell therapies [12]. The age, sex, and preexisting conditions of the patients also influence the extent of the regenerative potential [13]. The same is true for the extraction technique [14, 15] and the type of cell application. The source of the therapeutically used cells is discussed from various perspectives [16]. As a general rule, ASCs are obtained from the subcutaneous fat compartment, but it is also possible to isolate these cells from visceral tissue. Previous research has suggested that S-ASCs and V-ASCs have different properties [17]. Previous literature is inconsistent with respect to the therapeutic potential of stem cell populations from the subcutaneous and visceral compartments as well as differences in donor dependence [18]. With regard to the chondrogenic regenerative potential, for example, better functionality was shown when using subcutaneous tissue as a source for ASCs [9].

It is necessary to clarify the applicability of different stem cell populations as therapeutics for different indications. Therefore, the present work analyzes the gene expression profiles of murine ASCs from the subcutaneous (S-ASCs) and visceral (V-ASCs) compartments at single-cell level and compares these cells with regard to their therapeutic potential in an established wound healing model of the mouse [19].

## 2. Material and Methods

### 2.1. Funding

This work was supported by the German Research Foundation (DFG) and the Technical University of Munich (TUM) in the framework of the Open Access Publishing Program.

### 2.2. Single-Cell Gene Expression Analysis

The S-ASCs and V-ASCs were obtained from the inguinal fat and omentum of 6 mice (C57BL/6, 3 months old, male) as previously described [6], all experiments being performed in accordance with the relevant guidelines and regulations. S-ASCs and V-ASCs were isolated from SVF (stromal vascular fraction) using the surface marker profile CD34+/CD73+/CD90+/CD105+ and CD45−/CD31− (to exclude hematopoietic and endothelial cell contamination). The cells were sorted as single cells using a Becton Dickinson FACSAria Flow Cytometer into a 96-well plate (one cell per well) with 6 *μ*l lysis buffer in each well. Reverse transcription and low cycle preamplification were performed using CellsDirect (Invitrogen) with TaqMan primer sets (Applied Biosystems) as prescribed by the manufacturer. cDNA was loaded on 96.96 Dynamic Arrays (Fluidigm, South San Francisco, CA) for qPCR amplification using Universal PCR Master Mix (Applied Biosystems) with a specific TaqMan assay primer set [20].

### 2.3. In Vivo Wound Model

12-week-old male C57Bl/6 mice were randomized into three treatment groups: with S-ASCs and V-ASCs seeded hydrogels (2 × 105 ASCs) and one unabsorbed hydrogel control (*n* = 5 per group). As previously described [21], two 6 mm sores were placed on the back of each mouse, with each of the wounds being kept open by suturing on silicone rings to prevent wound contractions. All wounds were occlusively dressed (Tegaderm, 3M, St. Paul, MN). Digital photographs were taken on days 0, 3, 5, 7, 11, and 14. The wound area was measured using ImageJ software (NIH, Bethesda, MD).

### 2.4. Immunohistochemistry (IHC)

Histological samples of the wounds were obtained after wound closure and immediately embedded in OCT (Sakura Finetek USA Inc.). To evaluate neoangiogenesis, seven-micron thin sections were immunohistochemically stained for CD31 (1°—1 : 100 Rb *α*CD31, Ab28364, Abcam; 2°—1 : 400 AF547 Gt *α* Rb, Life Technologies). Nuclei were stained with DAPI, and ImageJ Software (NIH, Bethesda, MD) was used to binarize the images. Intensity hurdles were used to quantify CD31 staining based on pixel positive areas per field.

### 2.5. Statistical Analysis

The results are presented as mean ± standard error (SEM). The data analysis was performed by Student's *t*-test. The results were considered significant from *p* ≤ 0.05. For the single-cell expression analysis, a two-sample Kolmogorov-Smirnov (K-S) test was used to compare the empirical distribution: a strict cutoff at *p* < 0.01 according to Bonferroni correction for several samples took place. Transcriptionally defined subpopulations were selected using an adaptive fuzzy c-means clustering algorithm and standard Euclidean distance metric as described previously [20]. Each cell was assigned a partiality to each cluster based on similarities in its expression profile, with optimally partitioned clusters averaged out and filtered by hierarchical clustering to simplify the visualization of data patterns within and across clusters. Canonical pathway calculations and network analyses were performed using Ingenuity Pathway Analysis (IPA, Ingenuity Systems, Redwood City, CA) based on significantly overexpressed genes in the respective cellular subpopulation.

## 3. Results

### 3.1. Single-Cell Gene Expression Analysis

**Figure 1:**
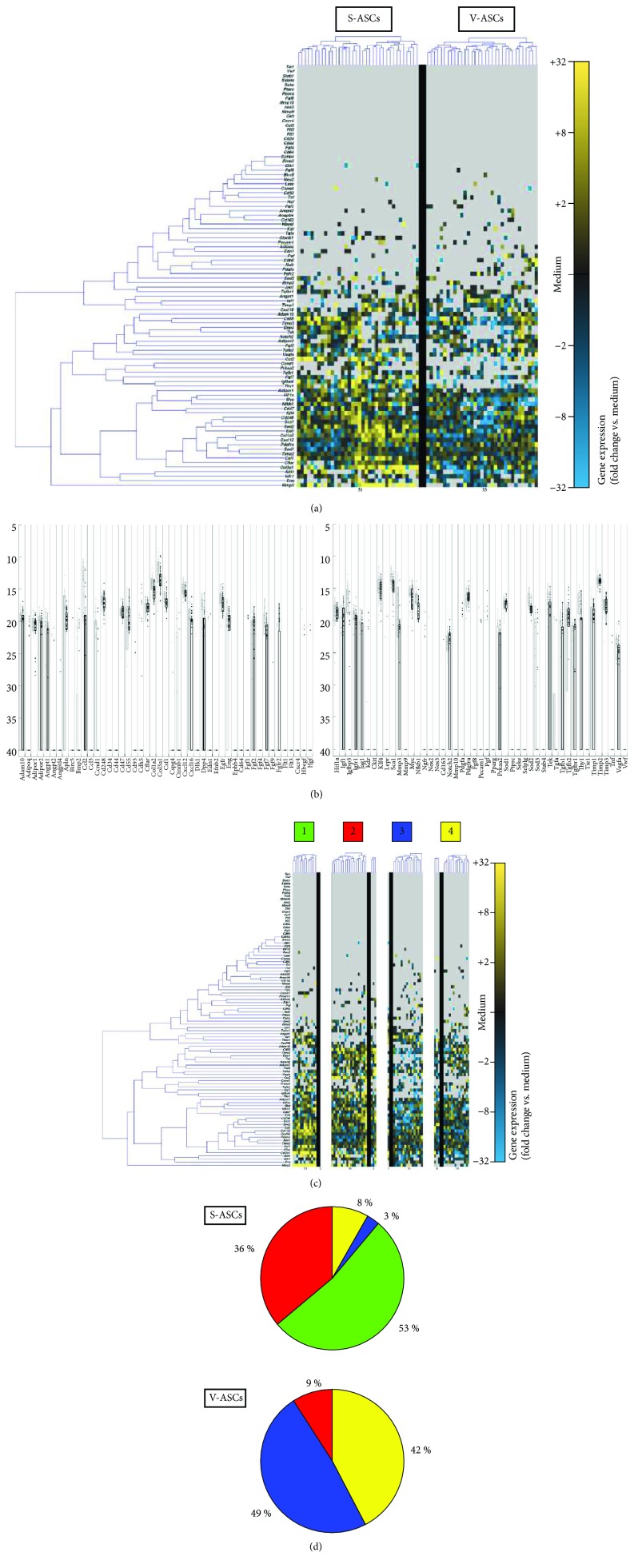
Single-cell transcriptional analysis of S-ASCs and V-ASCs. (a) At the single-cell level, S-ASCs and V-ASCs showed significant differences in genes related to neoangiogenesis and tissue remodeling (such as Ccl2, Hif1*α*, Fgf7, and Igf). Gene expression is shown on a color scale from yellow (high expression) to blue (low expression). (b) Whisker plots show qPCR cycle threshold for each gene. Individual dots each represent a cell/gene qPCR reaction, (c) clustering of S-ASCs and V-ASCs based on their gene expression patterns. (d) Pie charts represent the fractions of the ASCs that make up the respective clusters.

A gene expression analysis established in previous studies was used [22] to evaluate the transcriptional profile of single cells simultaneously for 96 genes in association with cell migration, proliferation, and tissue regeneration [17] (Table S1). At the single-cell level, S-ASCs and V-ASCs showed different transcriptional fingerprints. In particular, this is illustrated by significant differences in genes related to neoangiogenesis and tissue remodeling (such as Ccl2, Hif1*α*, Fgf7, and Igf) (Figures 1(a) and 1(b)).

Single cells can be reliably grouped by a clustering algorithm according to their gene expression profile [23]. To further investigate the differences in the expression profiles of S-ASCs and V-ASCs, an algorithm for identifying specific cellular subgroups was used [22]. As a result, four distinct subpopulation clusters were discovered (Figure 1(c)). While S-ASCs are mainly found in clusters 1 and 2, V-ASCs concentrated in clusters 3 and 4 (Figure 1(d)).

**Figure 2:**
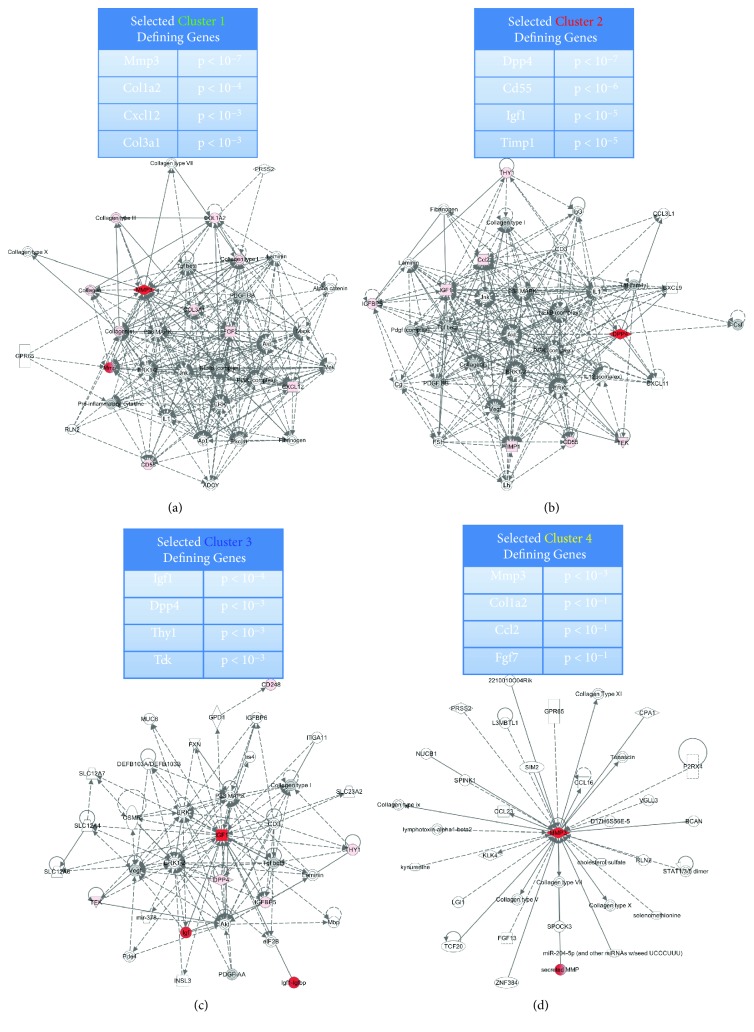
Analysis of ASC subpopulation. ((a–d), above) Significantly increased expressed genes of the individual clusters. ((a–d), below) Top Ingenuity Pathway Analysis (IPA) transcriptome networks based on the significantly overexpressed genes of clusters. These significant genes are shown in red.

The cluster classification further sheds light on how the origin of the subcutaneous or visceral compartment influences certain key signaling pathways at the cellular level. Using highly overexpressed cluster genes as a source, canonical signaling pathways were queried by Ingenuity Pathway Analysis (IPA, QIAGEN, Redwood City, CA, http://www.qiagen.com/ingenuity). Clusters 1 and 2 are characterized by significant overexpression of genes responsible for extracellular matrix remodeling and cell recruitment (for example, matrix metalloproteinase 3 (Mmp3; *p* < 10^−7^), collagen 1a2 (Col1a2; *p* < 10^−4^), and stromal cell factor 1 (Cxcl12/Sdf1; *p* < 10^−3^) (Figure 2(a)) as well T cell and complement activation (dipeptyl-peptide4 (Dpp4/Cd26; *p* < 10^−7^) and Cd55; *p* < 10^−6^) (Figure 2(b)). In contrast, cluster 3 is characterized by significantly increased expression of other trophic factors, such as insulin-like growth factor 1 (Igf1; *p* < 10^−4^) (Figure 2(c)). Cluster 4 is rather inexplicitly defined by Mmp3, *p* < 10^−1^ (Figure 2(d)).

### 3.2. In Vivo Wound Healing Model

**Figure 3:**
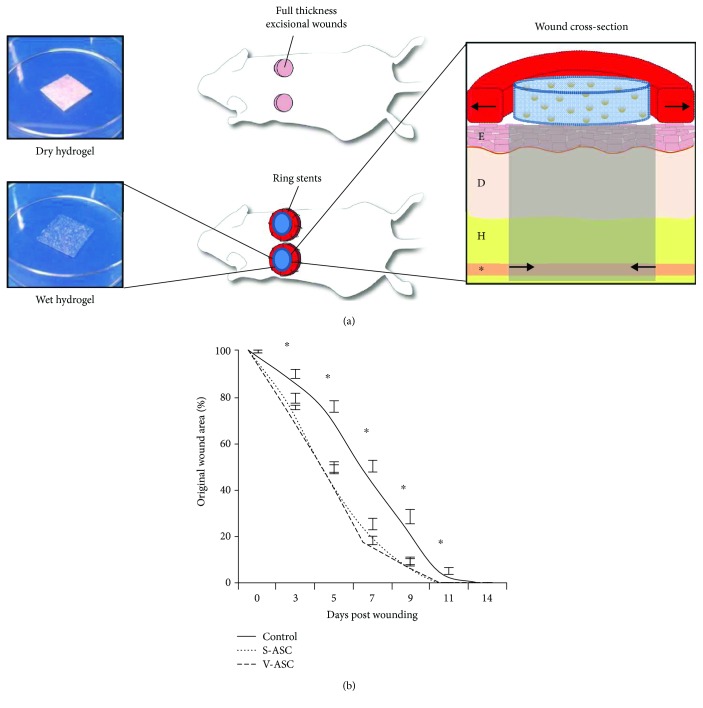
The application of both S-ASCs and V-ASCs improves wound healing (time to complete reepithelialization) in an in vivo model. (a) An illustrative schematic of the wound healing model showing the hydrogel scaffolds on the left and the mouse wounds in detail on the right. E, epidermis; D, dermis; H, hypodermis; asterisk, panniculus carnosus. Modified with permission from “The Journal of Investigative Dermatology” and the Journal “Plastic and Reconstructive Surgery.” (b) Wound area was measured at days 3, 5, 7, 9, 11, and 14. Both S-ASC- and V-ASC-treated wounds showed significantly enhanced wound closure at each time point (^∗^ *p* ≤ 0.05).

**Figure 4:**
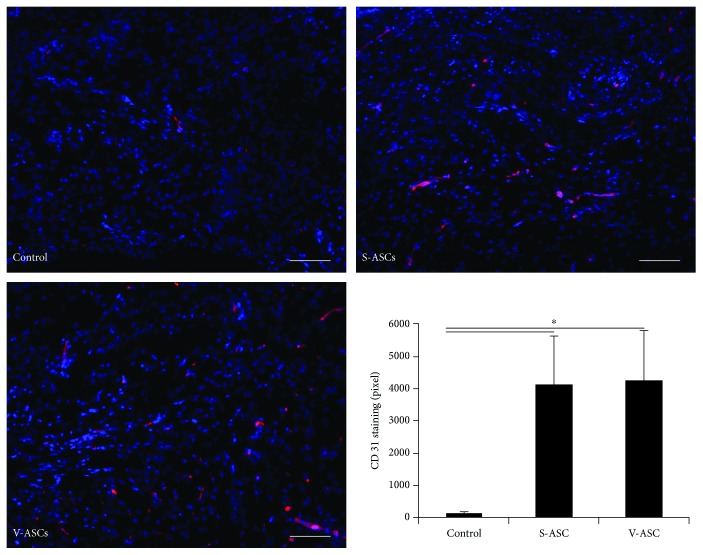
Immunohistochemical staining of the healed wounds shows comparable neovascularization by S-ASCs and V-ASCs. We were able to show comparable neovascularization (CD31 staining) induced by S-ASCs and V-ASCs. When comparing S-ASC- and V-ASC-treated wounds with control group, we found significantly enhanced CD31 levels. Scale = 25 *μ*m (^∗^ *p* ≤ 0.05).

Having demonstrated that S-ASCs and V-ASCs represent heterogeneous cell populations and that profound differences in the distribution of stem cell subpopulations exist between these tissue sources, we next investigated to what extent the origin of ASCs affects tissue repair in vivo. Despite marked differences in gene expression profiles, the use of S-ASCs and V-ASCs alike resulted in an accelerated healing rate in an established murine wound healing model compared to the control group (10.4 days to wound closure compared to 11.9 days; *p* < 0.05) without significant differences between treatment groups (Figure 3). The histological evaluation of the neoangiogenesis induction in the healed wounds also showed comparable results for both cell types and a significantly increased neoangiogenesis compared to the control group without cells (Figure 4).

## 4. Discussion

Stem cell therapy for the catalysis of healing processes has been extensively studied and described as a pathway into the future [24, 25]. In particular, the use of fat tissue-based stromal cells to accelerate wound healing has been extensively examined [26, 27]. With regard to the chondrogenic potential, the literature shows a better functionality when using subcutaneous adipose tissue [9]. Differences in terms of proliferation and colony-forming capacity are described with regard to cardiac muscle function after infarction and subsequent treatment with subcutaneous and visceral ASCs, but a positive effect on myocardial contractility without significant difference was found for both treatment groups [5].

V-ASCs showed an increased expression of gene clusters with respect to lipid biosynthesis and metabolism. These differences were thus dependent on the provenance of the cells. In contrast to the present study, the V-ASCs and S-ASCs were previously often examined in bulk by means of microarray or PCR, which, however, is inferior to the granularity of the single-cell method described herein [17].

Neovascularization as an important mechanism for the promotion of wound healing by ASCs has been scientifically described. The paracrine secretion of numerous addition, an increase in both recruitment and functionality of progenitor cells from the blood to the wound, could be demonstrated by ASC therapy [6]. The model of the mouse wound for the evaluation of cell therapeutic effects has long been established [28], and the positive effect of ASCs on wound healing has already been demonstrated several times. The ASC therapy was able to positively influence wound healing in the murine model even in the context of diabetes mellitus type II and obesity [29]. To date, however, there is still a lack of studies with a specific focus on the possible influence of the cell harvesting site on the treatment success [30].

The data presented here show that both subcutaneous and visceral ASCs have a positive influence on wound healing. Despite comparable therapeutic potential, gene expression of V-ASCs and S-ASCs is quite different. Through a cluster model, different cell subpopulations could be detected, which differed by their genetic fingerprint. Cluster 2 in particular comprises a selection of highly potent, regenerative genes and, with its surface marker profile (CD26/CD55), is comparable to a particularly functional cell population already described [12]. This cluster is mainly associated with S-ASCs. Interestingly, the visceral reservoir for wound healing also offers a relevant alternative. This suggests that more than the 96 genes we studied might influence wound healing. Our results also show that no clear indication of the functionality of cells can be derived from the genetic fingerprint alone. To correlate gene expression results with in vivo data is essential to verify the functionality and utility of the respective cells.

Further studies are needed to explore in detail the impact of gene expression on wound healing in terms of interacting proteins. Further investigations must analyze the specific cytokines and their effect on the local wound environment in detail. While there have been demonstrated large differences in chondrogenic healing [9], we are unable to show differences in wound healing comparing S-ASCs and V-ASCs. This further suggests that specific cell therapeutic indications require independent preclinical evaluations for cell therapy effectiveness.

## 5. Conclusion

The present study shows a high degree of heterogeneity in the gene expression profile of ASCs from the subcutaneous compartment compared to the visceral compartment. With similar therapeutic potential in the wound healing model, the significantly different gene expression patterns of ASCs suggest different mechanistic signaling pathways. This study clearly demonstrates that review of transcriptional results in vivo is advisable to functionally confirm potential effects of cell therapies.

## Data Availability

The data used to support the findings of this study are available from the corresponding author upon request.

## Supplementary Materials

Supplementary Materials Table S1: gene names and assay IDs for microfluidic single-cell gene expression analysis.
